# iElectrodes: A Comprehensive Open-Source Toolbox for Depth and Subdural Grid Electrode Localization

**DOI:** 10.3389/fninf.2017.00014

**Published:** 2017-03-02

**Authors:** Alejandro O. Blenkmann, Holly N. Phillips, Juan P. Princich, James B. Rowe, Tristan A. Bekinschtein, Carlos H. Muravchik, Silvia Kochen

**Affiliations:** ^1^FRONT Neurolab, Department of Psychology, University of OsloOslo, Norway; ^2^Estudios de Neurociencias y Sistemas Complejos, CONICET- El Cruce Hospital - Universidad Nacional Arturo JauretcheBuenos Aires, Argentina; ^3^Institute of Cellular Biology and Neuroscience “Prof E. De Robertis,” School of Medicine, University of Buenos Aires – CONICETBuenos Aires, Argentina; ^4^Epilepsy Section, Division of Neurology, Ramos Mejía HospitalBuenos Aires, Argentina; ^5^Department of Clinical Neurosciences, University of CambridgeCambridge, UK; ^6^MRC Cognition and Brain Sciences UnitCambridge, UK; ^7^Department of Psychology, University of CambridgeCambridge, UK; ^8^Facultad de Ingeniería, Instituto de Investigaciones en Electrónica, Control y Procesamiento de Señales, Universidad Nacional de La PlataLa Plata, Argentina

**Keywords:** SEEG, ECoG, intracranial EEG, MRI, CT, atlas, epilepsy

## Abstract

The localization of intracranial electrodes is a fundamental step in the analysis of invasive electroencephalography (EEG) recordings in research and clinical practice. The conclusions reached from the analysis of these recordings rely on the accuracy of electrode localization in relationship to brain anatomy. However, currently available techniques for localizing electrodes from magnetic resonance (MR) and/or computerized tomography (CT) images are time consuming and/or limited to particular electrode types or shapes. Here we present iElectrodes, an open-source toolbox that provides robust and accurate semi-automatic localization of both subdural grids and depth electrodes. Using pre- and post-implantation images, the method takes 2–3 min to localize the coordinates in each electrode array and automatically number the electrodes. The proposed pre-processing pipeline allows one to work in a normalized space and to automatically obtain anatomical labels of the localized electrodes without neuroimaging experts. We validated the method with data from 22 patients implanted with a total of 1,242 electrodes. We show that localization distances were within 0.56 mm of those achieved by experienced manual evaluators. iElectrodes provided additional advantages in terms of robustness (even with severe perioperative cerebral distortions), speed (less than half the operator time compared to expert manual localization), simplicity, utility across multiple electrode types (surface and depth electrodes) and all brain regions.

## Introduction

Human intracranial EEG (iEEG) recordings provide a unique contribution to cognitive neuroscience, allowing brain activity to be measured with high spatial and temporal resolution (Mukamel and Fried, 2012). Typically, subdural grids and depth electrodes are implanted in epilepsy patients undergoing pre-surgical evaluation, to characterize the areas involved in the genesis and propagation of seizures, i.e., the epileptogenic zone and eloquent cortex (Chauvel et al., 1987; Rosenow and Lüders, 2001; Kochen et al., 2002).

The spatial precision of iEEG recordings relies on accurate localization in relationship to brain anatomy (Lachaux et al., 2003). In clinical practice, accurate and robust localization of electrodes is essential for identifying the epileptogenic zone for resection (Pieters et al., 2013; Princich et al., 2013; Taimouri et al., 2014) and relating these findings to previous cognitive function tests, structural lesions, and other neuroimaging studies which are typically expressed in a standard anatomic space (McGonigal et al., 2007; Princich et al., 2013). In research, localizing electrodes within a standardized brain space also allows comparisons across subjects for group-level analysis (Keller et al., 2011; Kadipasaoglu et al., 2014) and enables direct comparison of single patient recordings to non-invasive imaging studies (Chennu et al., 2013; Phillips et al., 2016).

Many methods have been developed to locate implanted electrodes. Though some methods solely use MRI images to identify electrode locations (Kovalev et al., 2005; Ball et al., 2009; Gaillard et al., 2009; Axmacher et al., 2010; Yang et al., 2012), most methods also incorporate post-implantation CT images to obtain greater localization accuracy (Bootsveld et al., 1994; Van Rooijen et al., 2013). Currently available methods suffer from significant limitations, which we aimed to address with a new semi-automatic method.

Previous studies that co-register pre- or post-implantation MRI with CT images, utilize the tissue contrasts in the MRI to identify the anatomical positions of the electrodes which are seen more clearly in the CT images (Lachaux et al., 2003). Commonly, electrode positions are manually identified through observation of the associated CT artifacts in 2D views or 3D rendered brain images (Winkler et al., 2000; Tao et al., 2009; Princich et al., 2013). Manual identification is widely used, especially in clinical practice, but it is time consuming to identify and record the anatomical location of every electrode and requires detailed knowledge of MRI based neuroanatomy (Princich et al., 2013). To overcome the former drawback, we previously proposed the use of a brain atlas to make anatomical labeling fast and simple (Princich et al., 2013).

Studies co-registering post-implantation CT images to pre-implantation MRI images usually lead to miss-localization of grid electrodes by up to 14 mm from the brain surface because of fluid build-up around implanted grids (Dalal et al., 2008). To address this problem, localized electrodes can be projected in an orthogonal direction to the brain surface (Hermes et al., 2010; Taimouri et al., 2014) or fitted to the closest brain surface using a constrained energy-minimization algorithm in a spring like grid (Dykstra et al., 2012). Alternatively, methods that localize grid electrodes on post-implantation MRI and CT images do not need corrections since both images contain the same tissue-deformation (Winkler et al., 2000; LaViolette et al., 2011b; Ibáñez et al., 2013; Azarion et al., 2014).

In studies using only depth electrodes there is typically less tissue deformation. In 66 patients undergoing deep brain stimulation, Elias et al. (2007) observed a mean displacement of less than 1 mm in anterior commissure and posterior commissure coordinates and 3.5 mm displacement in the frontal poles due to stereotactic procedures. Thus, it can be beneficial in these situations to use pre-implantation MRI images without electrode artifacts and coregister them to post-implantation CT images (Ekstrom et al., 2008; Princich et al., 2013; Arnulfo et al., 2015).

Other methods use intra-operative photographs, which are registered with a pre-implantation MRI to localize subdural grids visible in the craniotomy (Wellmer et al., 2002; Pieters et al., 2013). Dalal et al. (2008) took the further step of also using X-ray registered images to localize grids not visible in the photographs, however this procedure requires several hours to be completed.

The localization methods described above require the manual selection of individual electrodes in the CT, MRI, or photographs (Dalal et al., 2008; Hermes et al., 2010; Dykstra et al., 2012; Yang et al., 2012; Pieters et al., 2013). Manual procedures are time consuming and prone to operator errors, thus semi-automatic and automatic localization methods have been developed, for example based on spatial filters (Sebastiano et al., 2006; Taimouri et al., 2014) or previous planning information (Arnulfo et al., 2015).

Most techniques are constrained to either localizing subdural grids or depth electrodes (Sebastiano et al., 2006; Dalal et al., 2008; Hermes et al., 2010; Yang et al., 2012; Pieters et al., 2013). However, it is not uncommon that specialized epilepsy centers use both subdural and depth electrodes in combination, in single patients, or in separate patients (Gonzalez-Martinez et al., 2013; Vadera et al., 2013; Enatsu et al., 2014). Moreover, the combination has increased in the last decade (Moshé et al., 2015) calling for a unified method of localization.

Here we present iElectrodes, a toolbox to localize both subdural grids and depth electrodes, and a pre-processing pipeline for MRI and CT images. The toolbox and pipeline were designed to meet the following objectives: usable in standardized anatomical space; speed; minimization of operator expertise dependency; and to be reliable across patients, locations, and electrode types. Additionally, we implemented an atlas-based anatomical description of each electrode location to facilitate interpretation and reporting and we developed an automated procedure to designate the electrode order. Finally, we made this toolbox an open-source application for the research and clinical community.

## Materials and methods

### Patients

Depth electrodes, grids or strips are commonly used in the clinical diagnosis protocol for the surgical treatment of drug-resistant epilepsy patients (Kochen et al., 2002; Kwan et al., 2010). We prospectively recruited 22 consecutive patients (7 female, mean age 29 years) with drug-resistant epilepsy, who were candidates for respective surgery. Intracranial electrodes were temporarily implanted for 5–10 days while iEEG and video were continuously recorded. MRI and CT images were acquired as part of the clinical procedure. The pre-surgical evaluation aimed to localize the epileptogenic zone and to delineate eloquent cortex (Rosenow and Lüders, 2001). Three patients were implanted with subdural grids, nine with subdural grids and depth electrodes, and eleven were exclusively implanted with depth electrode arrays (patients numbered from 1 to 3, 4 to 11, and 12 to 22 respectively). The study was conducted with the approval of the Research Ethics Committee of Ramos Mejía Hospital and El Cruce Hospital in accordance with the ethical standards laid down in the 1964 Declaration of Helsinki. All patients gave their written informed consent for the participation in this study and the use of their collected information.

### Electrode arrays

Cortical surface grids array dimensions were 2 × 4, 2 × 8, 4 × 5, 4 × 8, 6 × 8, and 8 × 8 contacts. Electrode strip dimensions were 1 × 4 contacts. Electrode contacts were made of platinum embedded in a 0.5 mm flexible silicon plate, with 4 mm diameter, 2.3 mm exposure diameter and 10 mm inter-contact center to center spacing (Ad-Tech Medical Instrument Corporation, USA). In some cases, a platinum marker was present in between contacts 1 and 2.

Depth electrode arrays had: (a) 8 or 10 platinum contacts with 5 or 10 mm inter-contact center to center distance, contact length of 2.4 mm and 1.1 mm diameter, or (b) 9 platinum contacts, 3 mm distance between the first and the second contact and 6 mm inter-electrode distance from the second to the last. Contact length was 1.57 mm and the electrode diameter was 1.28 mm. Simultaneously, 9 micro-wire platinum electrodes were implanted through the lumen of the electrode array. Each micro-wire had a diameter of 38 μm and was trimmed at ~4 mm past the tip (Ad-Tech Medical Instrument Corporation, USA).

The implantation sites were solely based on clinical criteria and had no relationship to the current study. Table 1 shows electrode implantation descriptions and anatomical locations for each patient. Due to the different targets achieved with deep electrode arrays, in some cases not all contacts were placed inside the brain and some remained in the skull or cerebrospinal fluid. Since those contacts were not electrophysiologically relevant, they were excluded from the current study.

**Table 1:** **Implantation details**.

**Patient no**.	**Gender**	**Age**	**Subdural grids**			**Depth electrodes**			**Brain areas**					
			**Hem**	**N**	**Arrays**	**Hem**	**N**	**Arrays**	**F**	**T**	**P**	**O**	**I**	**L**
1	F	20	R	64	(8 × 8)					✓	✓	✓		
2	M	33	L	64	(8 × 8)				✓	✓	✓			
3	M	23	R	48	(4 × 8); (2 × 4); 4; 4				✓					
4	M	59	L	64	(8 × 8)	L/R	20	10/10	✓	✓	✓	✓		✓
5	M	29	L	64	(4 × 8); (4 × 8)	L	23	9; 9; 5	✓	✓	✓			
6	M	44	R	24	(4 × 5); 4	L/R	20	10/10	✓	✓		✓		✓
7	M	29	R	28	(4 × 5); 4; 4	R	20	10; 10	✓	✓		✓		✓
8	F	30	L	48	(6 × 8)	L	29	10; 5; 5; 9	✓	✓	✓	✓		✓
9	M	20	R	104	(8 × 8); (2 × 8); (2 × 8); 4; 4	R	8	4; 4	✓	✓	✓	✓		
10	F	44	L	52	(4 × 8); 4; 4; 4; 4; 4	L	10	5; 5	✓	✓				✓
11	F	24	L	52	(6 × 8); 4	L	12	6; 6	✓		✓			
12	M	26				L	31	9; 9; 7; 6	✓	✓	✓		✓	
13	M	20				L/R	23	3; 7; 6; 2/5		✓		✓		✓
14	F	22				R	44	9; 9; 8; 9; 9	✓	✓			✓	✓
15	M	19				L/R	47	7; 8; 8/7; 8; 9		✓				✓
16	F	49				L/R	56	5/7; 6; 9; 6; 3; 7; 7; 6		✓	✓	✓		✓
17	M	19				L	36	7; 7; 7; 7; 8	✓	✓				✓
18	M	21				L/R	47	8; 8; 8/8; 7; 8		✓				✓
19	M	25				R	46	7; 7; 7; 9; 7; 9		✓		✓		✓
20	M	37				L/R	48	8; 8; 7/8; 8; 8		✓				✓
21	M	33				L	42	7; 10; 7; 4; 7; 7	✓	✓	✓			✓
22	F	23				R	68	9; 10; 7; 10; 8; 10; 7; 7	✓	✓	✓			✓
Average (total)	7F/15M	29.5	6L/5R	55.6 (612)		7L/5R /7B	33.2 (630)		68% (15)	90% (20)	50% (11)	40% (9)	9% (2)	68% (15)

*Array dimensions (cols. 6 and 9) are separated by semicolons. Left and right hemisphere implantations are separated by slash. Hem, Implantation hemisphere; N, number of implanted electrodes; F (top row), frontal lobe; T, temporal lobe; P, parietal lobe; O, occipital lobe; I, insular cortex; L, limbic lobe; F (other rows), female; M, male; L, left hemisphere; R, right hemisphere*.

### Acquisition of MRI and CT images

T1-weighted MRI FFE sequence with 1 mm isotropic resolution images were acquired within 2 days after implantation in patients with subdural grids (with or without depth electrodes, patients 1–11, Achieva, Phillips Medical Systems, The Netherlands, 1.5 T magnet unit, TR/TE/TI = 9.2/4.2/450 ms, matrix 256 × 256, FOV 256 × 256 mm, slice thickness 1 mm, and 175 slices), and prior to implantation in patients with depth electrodes only (patients 12–22, Achieva, Phillips Medical Systems, The Netherlands, 3 T magnet unit, TR/TE = 6.9/3.2 ms, matrix 256 × 256, FOV 256 × 256 mm, slice thickness 1 mm, and 180 slices). Special care was taken when acquiring MRI images with intracranial electrodes. Each multi-channel connection lead was keep straight and without touching any other lead, forming no loops which could potentially induce currents (Davis et al., 1999). MRI of implanted patients has been shown to be safe, with respect to possible movements induced by electromagnetic fields and heating of electrodes (Davis et al., 1999; Carmichael et al., 2008; Princich et al., 2013; Azarion et al., 2014).

CT scans for each patient were performed within 2 days after electrode implantation to visualize electrode locations and as part of the clinical protocol for the evaluation of possible complications such as hematoma, contusions or subdural effusions. CT images (Emotion, Siemens Medical Solutions, Germany, 120 kVp, 240 mm FOV, 512 × 512 matrix, 0.6 mm slice thickness for patients 1–11, or Aquilion, Toshiba Medical Systems, Japan, 120 kVp, 220–230 mm FOV, 512 × 512 matrix and 0.5 mm slice thickness for patients 12–22) were reconstructed to isotropic 1 mm resolution.

### Image pre-processing

**Figure 1:**
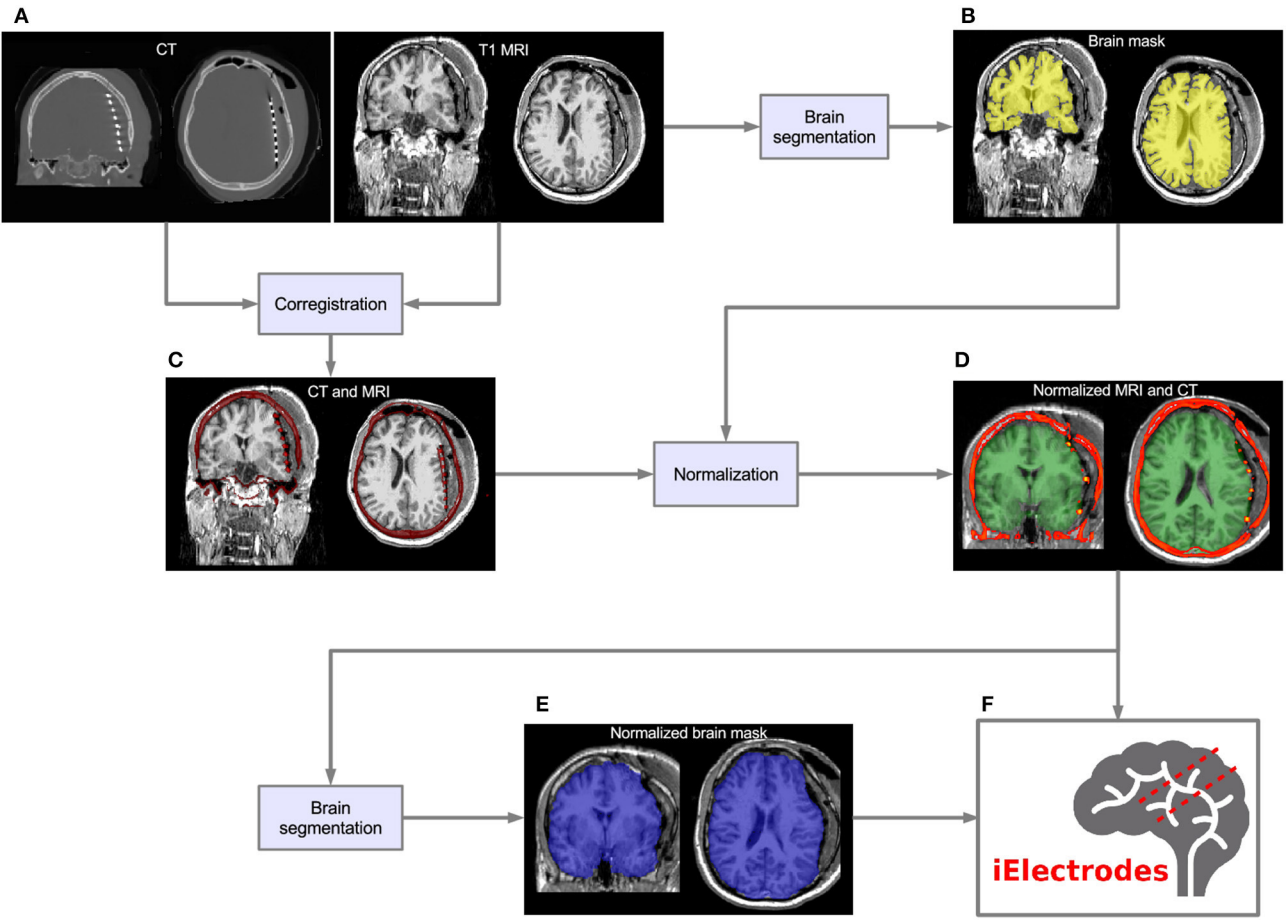
**Pre-processing pipeline. (A)** Acquired images, CT and T1 MRI, showing electrode artifacts, brain shift and compression caused by edema. **(B)** A brain mask (yellow) is obtained by segmenting the MRI. Notice that the mask border follows the brain surface accurately. **(C)** MRI and CT images are coregistered in native space. Observe the electrode artifacts in the thresholded CT (red) over the MRI. **(D)** Using the previous brain mask (yellow), the MRI is spatially normalized and the same transformation applied to the CT. Observe the CT (red) on top of the normalized brain MRI. For illustrative purposes we also show the MNI average brain mask (green). **(E)** A normalized brain mask is obtained (blue) from the normalized MRI. **(F)** Normalized MRI, CT, and brain mask images are loaded into the iElectrodes toolbox. Example images correspond to patient 2, implanted with an 8 × 8 grid over the left frontal, temporal, and parietal lobes.

Figure 1 shows an overview of the pre-processing pipeline. The acquired MRI and CT images were exported in DICOM format and then transformed to the NIfTI standard using dcm2nii software (MRIcron, USA). Pre-processing requires the coregistration of the images before they can be processed using the iElectrodes toolbox. We also normalized the images to a standard space as part of the pre-processing pipeline. However, this step can be omitted if a native space representation is required, as it is in some clinical practice routines (Princich et al., 2013).

Although we have implemented the pre-processing steps using a particular set of image analysis softwares, pre-processing can be performed using other available packages. The ability to adopt alternative normalization procedures will be of particular interest, in view of the marked peri-operative cerebral deformations arising with subdural grids.

#### MRI cortical segmentation

We performed subject-specific cortical segmentation using the Freesurfer v5.0 image analysis suite (Dale and Sereno, 1993; Dale et al., 1999), obtaining a segmented image of the cerebral cortex, based on gyral and sulcal structures (Figure 1B; Fischl et al., 2004; Desikan et al., 2006).

#### MRI and CT coregistration

CT images were coregistered to MRI images (Figure 1C) in SPM8 toolbox (Wellcome Trust Centre for Neuroimaging, UCL) using a 6-parameter rigid-body transformation based on the maximization of the normalized mutual information (Studholme et al., 1998), which has shown to perform well for the coregistration of MRI and CT image modalities (Ken et al., 2007; Hermes et al., 2010; Azarion et al., 2014). Coregistered images were visually checked.

#### Spatial normalization

MRI images were normalized to the Montreal Neurological Institute (MNI152) standard space in SPM8 (Figure 1D). Brain masks obtained from Freesurfer were used in the normalization procedure to avoid unwanted deformations due to non-standard tissue (see Supplementary Figures 1A,B). Normalization was performed in two steps: (a) bias correction and (b) non-linear coregistration (Ashburner and Friston, 1999). This process includes non-linear warping transformations to account for the large deformations observed due to the surgical procedure (see Figure 1A). The same non-linear transformation was applied to the coregistered CT images (Figure 1D). Other normalization procedures may be adopted for use with iElectrodes.

#### Brain segmentation of normalized brain

Brain segmentation masks were obtained from normalized MRI images using FMRIB software library (FSL, Oxford, UK) Brain Extraction Tool (BET) (Smith, 2002; Jenkinson et al., 2005; Figure 1E). These brain masks were useful in circumscribing the regions where intracranial electrodes were localized.

### Localization of intracranial electrodes in iElectrodes toolbox

Coregistered and normalized MRI and CT images, as well as brain masks, were loaded into the iElectrodes toolbox in NIfTI format. The toolbox was implemented in MATLAB (The Mathworks Inc., USA) using a graphical user interface, and includes in-house designed algorithms and functions from other publicly available MATLAB toolboxes (see details in the acknowledgments). The sequence of steps to localize, number, and label all electrodes is described below. In brief, all voxels corresponding to intracranial electrodes were obtained by masking and thresholding the CT. Then, electrodes coordinates were determined as the center of mass of each cluster of voxels. Finally, electrodes were numbered and anatomical labels assigned to each one using a probabilistic atlas. From the user perspective, the localization procedure is fast and straightforward. Users are required to manually determine the brain mask size and the threshold value, and select all voxels corresponding to a single array. Finally the user must run the automatic localization, numbering, and anatomical labeling algorithms. Two sample videos demonstrate the localization procedure: Supplementary Video 1 (subdural grid) and Supplementary Video 2 (depth electrodes).

#### Masking and thresholding

**Figure 2:**
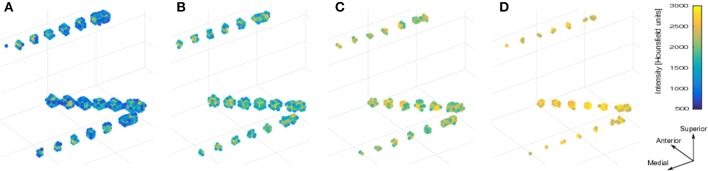
**Voxels thresholding**. The use of different threshold values on the CT produces more diffuse or clear views of the electrodes. **(A)** A low threshold value (700 HU) resulted in electrodes appearing to be merged together. **(B,C)** Middle threshold values (1,200 HU and 1,700 HU) resulted in a cleaner distinction between electrodes. **(D)** A high threshold value (2,200 HU) resulted in a few voxels per electrode. Notice that the two medial (deeper) contacts in each array are closer (3 mm) than all other contacts, making its visual differentiation more difficult. Example data from patient 17 showing one frontal and two temporal depth electrode arrays. HU, Hounsfield Units.

CT voxel intensities that corresponded to electrodes were higher than most of the head tissue intensities, with the exception of dense bone within the skull. The normalized brain mask was dilated and/or eroded using a cubical nucleus of 3 × 3 × 3 voxels, removing the unwanted CT high intensity signal from the skull. The number of erosion or dilation iterations of the brain mask was adjusted according to the subject and electrode array needs. Simultaneously, a voxel intensity threshold was set to 1,800 Hounsfield units and then adjusted manually to visualize the voxel clusters corresponding to the electrodes without brain tissue. If the threshold was too low, it was not possible to distinguish one electrode from its neighbors. If the threshold was too high, electrode voxels were not well defined. Figure 2 shows the effect of different thresholding values. The threshold values were adjusted for each electrode array, to account for differences in intensity levels. Spurious non-electrode voxels were manually removed if necessary, for example when the connection cable from one depth electrode crosses over a grid, or the removal of the metallic markers located between the first two contacts within some grids.

#### Voxel selection, clustering, and localization

**Figure 3:**
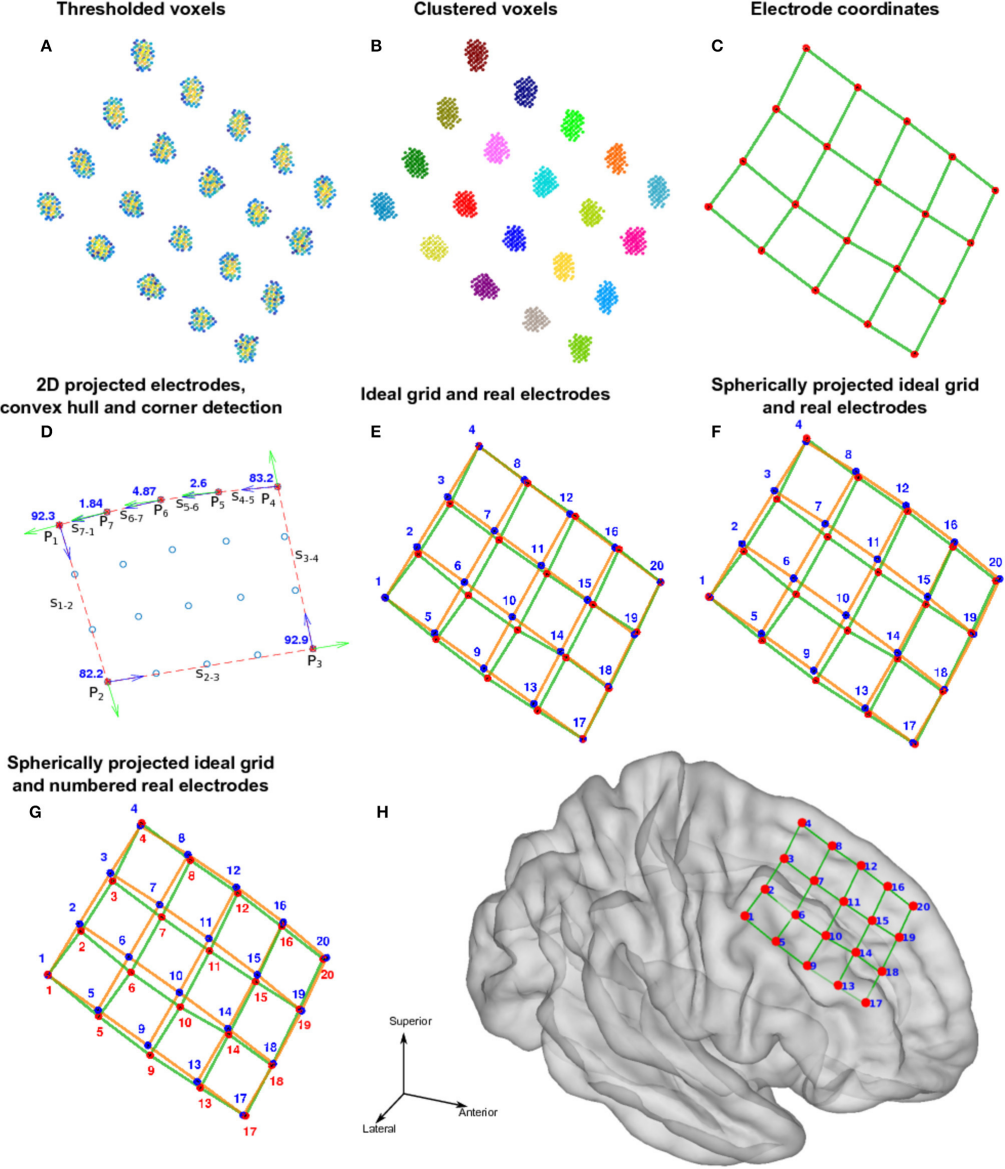
**Localization and numbering of electrodes. (A)** Thresholded CT voxels corresponding to a 4 × 5 grid (color coded HU) from patient 6. **(B)** Clustered voxels (each color denotes a different cluster). **(C)** Detected electrodes locations (red dots), i.e., center of mass of each cluster. **(D)** Electrode coordinates projected in 2D principal components space (blue circles). Convex hull described by a subset of points P (red asterisks) and segments S (red lines) connecting this points. Arrows show previous (green) and next (blue) segment direction at each point. The transition angle (in degrees) at each point is shown (blue). Points 1, 2, 3, and 4 correspond to the four biggest angles and also the corners of the grid. **(E)** Ideal planar grid (blue dots) and real grid electrode locations. Ideal grid electrodes are numbered (blue). **(F)** Radial projection of ideal electrode grids. **(G)** Real grid electrodes are numbered (red) according to closest ideal grid electrodes. **(H)** MNI semitransparent brain with localized and numbered electrodes.

We used the selection drawing tool to select the electrode voxels for each electrode array in a 3D space (Figure 3A). This procedure is facilitated by rotations, zoom, and pan in the 3D space plot. After selection, all electrode voxels (N voxels) were clustered using a K-means algorithm (Hartigan and Wong, 1979; Figure 3B). In this step, K is the number of electrodes within each electrode array, which was obtained from implantation notes. Briefly, the K-means algorithm divides the N voxels into K clusters, whilst minimizing the within-cluster sum of squares. Then, the center of mass coordinate L_*k*_ of each electrode (k = 1,2,…,K) is calculated as a weighted average of all voxel coordinates inside each cluster, using the voxels signal intensities as weights (Figure 3C). A matrix L, of dimension Kx3, was constructed containing all electrode coordinates.

#### Automatic electrode numbering

**Figure 4:**
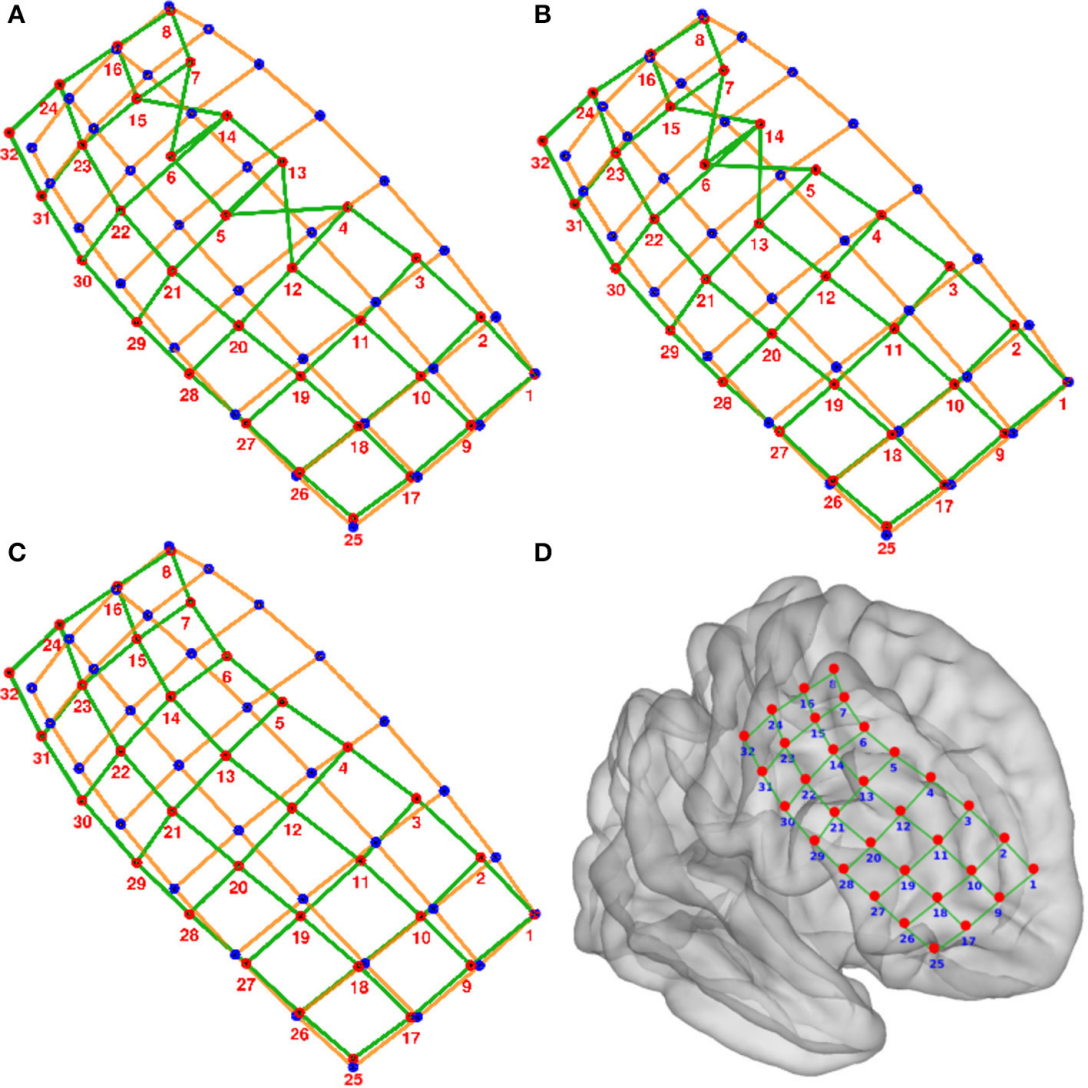
**Numbering of electrodes using permutation corrections. (A)** First numbering of electrodes based on searching the minimum distance between ideal (blue dots) and real (red dots) electrodes. **(B)** Electrode numbering after first permutation correction. **(C)** Electrode numbering after second (last) permutation correction. **(D)** MNI semi-transparent brain with localized and numbered electrodes. Connecting lines are for illustrative purposes only. Example grid obtained from patient 3.

Each electrode contact had a different name or label assigned to it, uniquely identify the recording signal from a particular site. For every array of electrodes, we divided the labeling procedure into two steps; first numbering the electrodes and then naming. The numbering procedure was different for depth electrodes and grids. Here, we the terms numbering and indexing equivalently. The following steps are illustrated in Figures 3, 4.

- Depth electrodes.
  1. Since these electrode arrays are well described in a unidimensional space, we projected the L_*k*_ coordinates onto the first component (maximum variance dimension) of the Principal Component Analysis (PCA) decomposition of L.
  2. Electrodes were automatically numbered according to their ordinal position in this dimension. Number 1 was assigned to the deepest electrode.

- Grid electrodes.
  1. We projected the grid coordinates L_*k*_ onto the first two principal components (2D space) of the PCA decomposition of L (Figure 3D).
  2. A convex hull was calculated from these coordinates in the 2D space. We obtained a subset of M points P_1_, P_2_, …, P_M-1_, P_M_ describing the convex hull and M segments S_1—2_, S_2—3_, …, S_M-2—M-1_, S_M-1—M_ connecting them (Figure 3D).
  3. We measured the angle at each segment transition, i.e., the angle between segments S_1—2_—S_2—3_, S_2—3_—S_3—4_, …, S_M-2—M-1_—S_M-1—M_, S_M-1—M_—S_M—1_ (Figure 3D). The grid corners were associated to the four largest transition angles, assuming that we were working on rectangular grids.
  4. An “ideal” grid was modeled using the corner coordinates, and the known number of rows and columns. This procedure estimated the expected coordinate of each electrode (Figure 3E). Corner electrodes in the real grid and ideal model correspond to the same point in space. We also specified electrode indexing numbers in the ideal array in accordance to its row and column position. Number 1 was assigned arbitrary to any of the possible corners coordinates.
  5. Real electrode coordinates lie in a curved surface, following the envelope of the brain contour (Figure 3C). Therefore, we radially projected the ideal grid coordinates to a spherical surface that best fits all real coordinates (Figure 3F).
  6. The electrode numbers were assigned to each real electrode according to the closest ideal electrode in terms of Euclidean distance (Figure 3G). Indices were assigned one by one, from short to long distances.
  7. The sum of distances between the real and ideal grids was minimized by permuting real grid indices (Figure 4):
    - All neighbor electrode indices were permuted, one location at the time, by pairs in the row and column dimension of the grids. The sum of the distances among the real and ideal grid electrodes was measured at each permutation.
    - If one of these sum of distances was smaller than the non-permuted one, the corresponding permutation was accepted and step (i) was repeated. If not, the loop was ended.
  8. The sum of distances between closest neighbors in the real grid was minimized following the same permutation procedure as in step 7.
  9. Manual flips (up, down, left or right) and rotations (clockwise or counter-clockwise) of electrode numbers were done in agreement with real order of electrodes using implantation planning diagrams.

Naming electrodes was the last step for both grid and strips. Each electrode in the array was named using a prefix—number concatenation, for example Grid1, Grid2, Grid3,…GridK in a grid of K electrodes, or an ordered list of labels obtained from the iEEG recording files.

#### Anatomical labeling

We used the Harvard—Oxford probabilistic atlas registered to the MNI152 space to assign anatomical labels to each electrode. This description is relevant to describe and compare activation areas across subjects. The atlas was obtained from the FMRIB Software Library v5.0 (FSL) (Jenkinson et al., 2012).

### Validation of electrode localization

We defined three validation procedures to quantify the degree of success of the proposed method: visual inspection of position and labeling, quantitative measures of localization error, and normalization performance.

#### Visual validation

**Figure 5:**
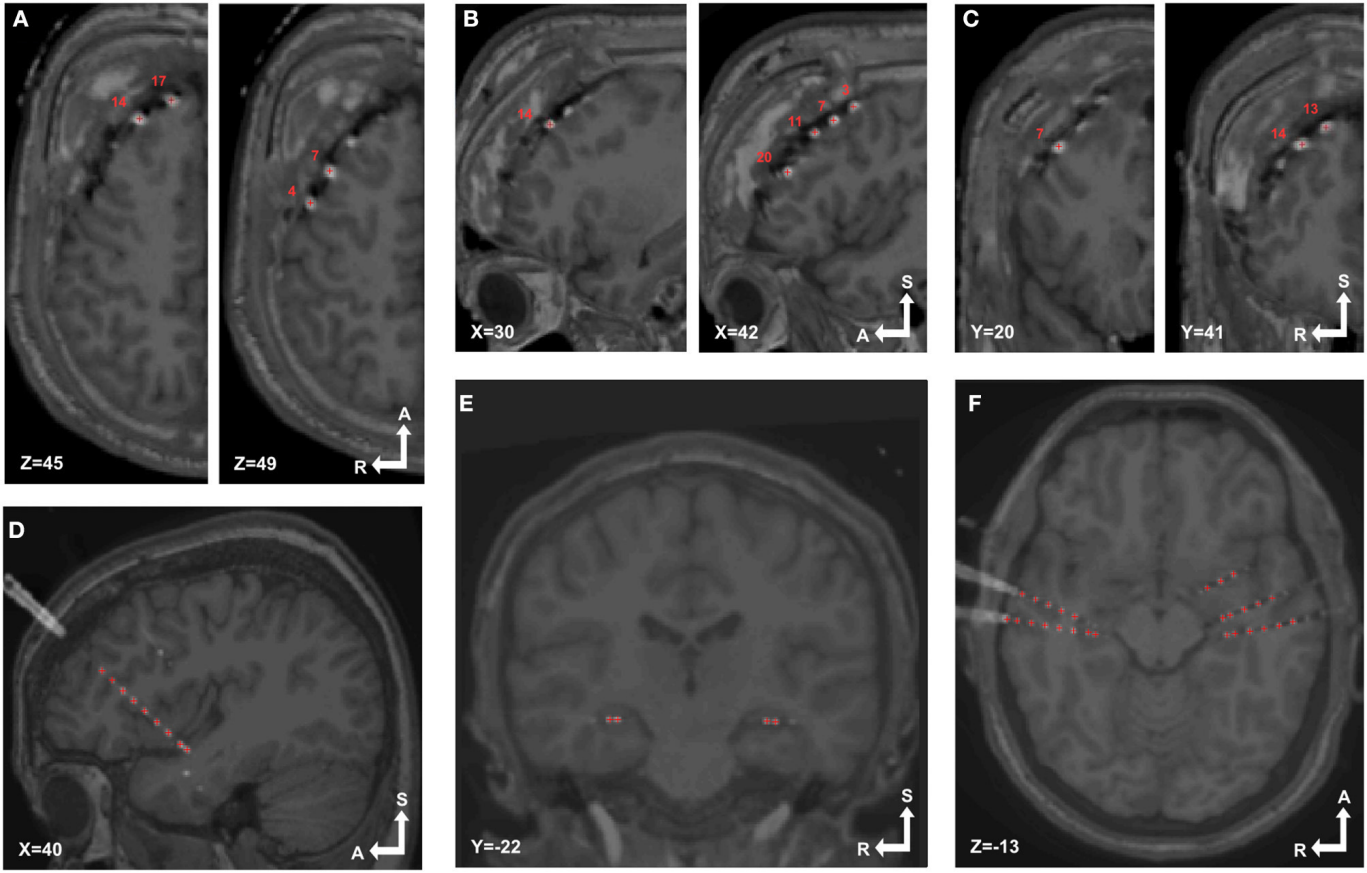
**Localized electrodes in MRI-CT**. MRI-CT blend images showing examples of electrode artifacts and detected coordinates (red crosses) superimposed. **(A–C)** Axial, sagittal and coronal views (respectively) of patient 6, implanted with a 4 × 5 grid over the right frontal lobe. **(D)** Sagittal view of patient 14 showing a depth electrode array in the insular cortex. **(E,F)** Coronal and sagittal view of patient 18 showing bilaterally implanted electrodes in hippocampi and temporal lobes.

Groups of electrodes in each array were visually checked in both 3D and 2D views. In 3D (Figures 3H, 4D) we checked that all electrodes were present and the labeling order was correct. Then we walked through all electrodes, one by one, and visually determined if the locations corresponded with the electrode artifact observed in the 2D orthogonal views of the fused MRI-CT images (Figures 5A–F).

#### Quantitative validation

A group of five independent experts were asked to manually and semi-automatically localize a subset of electrodes to validate the proposed localization procedure. The experts had 3–10 years of experience in electrode localization utilizing neuroimaging methods. The subset of electrodes was randomly chosen from five patients, two with subdural grids and three with deep electrodes. A maximum of 32 electrodes were localized in the patients implanted with grid arrays. Two randomly chosen arrays were localized in each of the patients implanted with depth electrodes. This criterion was formulated to validate the method with a representative sample of electrodes.

In the manual localization procedure, the experts were instructed to determine the location of each electrode center based on the 2D visualization of CT artifacts, using the three orthogonal views of CT images. They set image brightness and contrast settings to their most comfortable values. They were able to select each coordinate, compare them, and correct their choices as many times as they wanted to. We will denote these manual locations as $L_{k,r}^{m}$ for electrode k and expert r, being k = 1,2,…,K, r = 1,2,…,R, K the total number of electrodes, and R the number of experts. Note that in order to simplify the notation, we have omitted the *x, y*, and *z* components of each coordinate.

In the semi-automatic localization procedure, the group of experts localized the same subset of electrodes using the iElectrodes toolbox as previously described in Section Localization of Intracranial Electrodes in iElectrodes Toolbox. We will denote these semi-automatic locations as $L_{k,r}^{s}$ for electrode k and expert r.

Krippendorff's alpha (Krippendorff, 1970, 2004) was used as a measure of the inter-rater agreement of the localization procedures. Krippendorff's alpha has the capability to accept several raters and ordinal ratings (Hayes and Krippendorff, 2007). Alpha defines reliability scale points, being 1 for perfect reliability and 0 for the absence of reliability. Alpha values higher than 0.8 indicate that ratings are reliable (Krippendorff, 2004).

We defined the “gold standard” location G_k_ for each electrode k as the mean of the manually localized coordinates across all experts $G_{k}=1/R\overset{R}{\underset{r=1}{\sum }}L_{k,r}^{m}$.

Localization errors from using our semi-automatic method were calculated as the Euclidean distance of each electrode coordinate to the corresponding gold standard location $e_{k,r}^{s-G}=||L_{k,r}^{s}-G_{k}||$, and equivalently for the manual method as $e_{k,r}^{m-G}=||L_{k,r}^{m}-G_{k}||$ (Taimouri et al., 2014). Additionally, we used Krippendorff's alpha as a measure of the inter-rater agreement of the manual localization procedure, and therefore the reliability of the “gold standard.”

Non-parametric permutation test statistics were used to evaluate the differences between semi-automatic and manual localization errors ($e_{k,r}^{s-G}$ and $e_{k,r}^{m-G}$ respectively) (Nichols and Holmes, 2001). Localization errors for depth and grid electrodes were analyzed individually and combined together. This simple method does not depend on Gaussian distribution assumptions about the probability distribution of the data. The combined data from the manual and semi-automatic electrode locations underwent a random partition, and a *t*-test was calculated. This process was repeated 100,000 times to construct a *t*-value distribution under the null hypothesis of no difference between the two procedures. From the test statistic that was actually observed and the permutation distribution, we calculated the proportion of random partitions that resulted in a larger test statistic than the observed one. The resulting percentile is reported as a *p*-value (Maris and Oostenveld, 2007).

We assessed the robustness of the proposed method to thresholding, masking and voxel selection by the different experts. We calculated location V_k_ for each electrode k as the mean of the semi-automatically localized coordinates across all experts $V_{k}=1/R\overset{R}{\underset{r=1}{\sum }}L_{k,r}^{s}$. We measured the error distance between electrode locations obtained semi-automatically to their corresponding mean coordinate $e_{k,r}^{s-V}=||L_{k,r}^{s}-V_{k}||$. We quantified the error distribution by its mean and standard deviation. Additionally, we used Krippendorff's alpha as a measure of the inter-rater agreement of the semi-automatic localization procedure.

#### Projection to smoothed cortical envelope and normalization performance

Subdural grids were expected to be on top of the cortical envelope of the MNI brain after the normalization procedure. Therefore, a smoothed cortical envelope (SCE) was constructed to take into account electrodes sitting above sulci. An MNI brain mask image was obtained from the Harvard-Oxford atlas in FSL (HarvardOxford-sub-maxprob-thr25-1mm.nii file) (Jenkinson et al., 2012). The brain stem structure and the cerebellum were removed, and left and right hemispheres were split. The image was processed with the vol2surf function (“cgalsurf” method) in ISO2Mesh toolbox for MATLAB (Fang and Boas, 2009). A tetrahedral mesh surface of the brain cortex was obtained for each hemisphere, the so called SCE.

Grid electrodes were projected to this surface while minimizing an energy cost function that considered the electrodes' displacement and the deformation of a spring like grid connecting the electrodes (Dykstra et al., 2012):

$$\underset{k=1}{\sum^{K}}\|L_{k}-L_{k0}{\|}^{2}+\underset{i=1}{\sum^{K}}\underset{j=i+1}{\sum^{K}}a_{ij}{\left(d_{ij}-d_{ij0}\right)}^{2}$$

constrained to $\forall k,||L_{k}-s_{k}|{|}^{2}=0$, where L_k_ is the coordinate of electrode k, L_k0_ is the original coordinate for electrode k, d_ij_ is the distance between electrodes i and j, d_ij0_ is the original distance among the same pair of electrodes, a_ij_ is a parameter that take the value of 1 when electrodes i and j are neighbors and 0 otherwise, and s_k_ is the closest node in the SCE mesh to electrode k. The minimization procedure was implemented in MATLAB using the “fmincon” function. The projection vector for each grid electrode is D_k_ = L_k_-L_k0_.

When depth electrodes were implanted simultaneously to grids, a displacement field function was estimated based on the grid electrodes' projection (Taimouri et al., 2014). Translations were applied to the depth electrodes with variable strength according to their distance to the grid electrodes. We defined a weight function for each pair of grid electrode k and depth electrode j as:

$$w_{jk}=exp\left(-\frac{\|L_{j}-L_{k}{\|}^{2}}{\sigma_{R}^{2}}\right)$$

where σ_*R*_ is a regularization parameter.

Depth electrodes close to the brain geometrical center were less affected by the spatial normalization than the ones on the brain surface. Accordingly, we calculated a weight function to attenuate the deformation field with distance

$${w\prime }_{jk}=exp\left(-\frac{\|L_{j}-L_{k}{\|}^{2}}{\sigma_{D}^{2}}\right)$$

where 2σ_D_ was calculated as the mean distance of all grid electrodes to the center of mass of the SCE. Finally, the displacement vector for each depth electrode j was calculated as:

$$F_{j}=\frac{\sum_{k=1}^{K}w_{jk}{w\prime }_{jk}D_{k}}{\sum_{k=1}^{K}w_{jk}}$$

Supplementary Figure 2 shows a simulated example of the displacement field function.

The performance of the normalization process was assessed by measuring the projection distance of grid electrodes to the SCE || D_*k*_||, and the displacement distance of depth electrodes || F_*j*_||.

## Results

**Figure 6:**
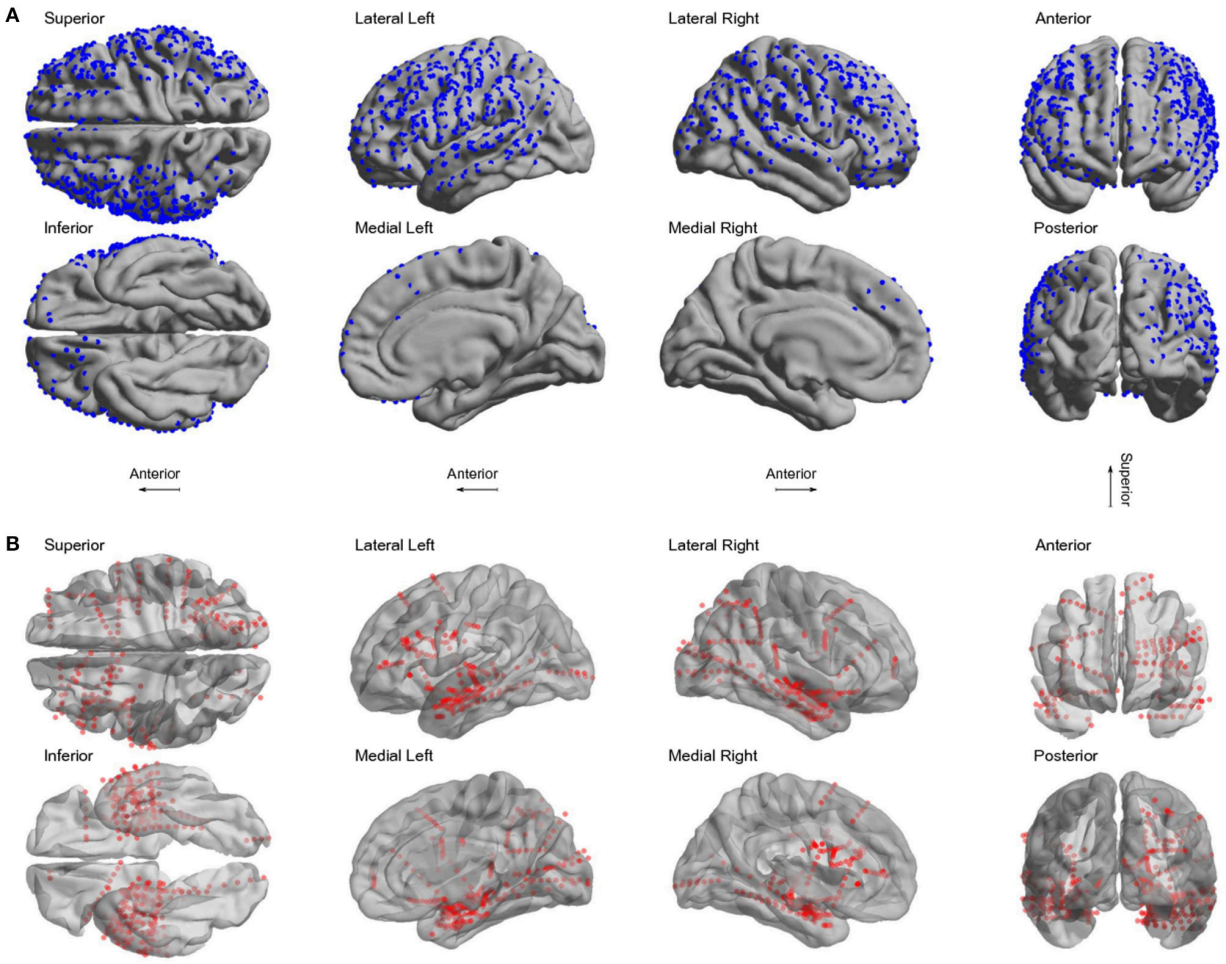
**Localized electrodes. (A)** Summary plot of all grid and strip electrodes projected over the brain surface in MNI space. **(B)** Summary plot of all depth electrodes are shown in the MNI semi-transparent brain. Only half of the brain surface and corresponding electrodes are shown in each view (Superior: z > 0, Inferior: z < 0, Left: x < 0, Right x > 0, Anterior: y > 0, Posterior: y < 0).

We localized 1,242 electrodes (612 grid and 630 depth) in 22 patients using the proposed method of pre-processing and the toolbox (see Table 1 for electrodes locations details). Electrodes were distributed across all brain lobes in both hemispheres. Figure 6 shows the electrode positions from all patients superimposed over a MNI standard brain.

**Figure 7:**
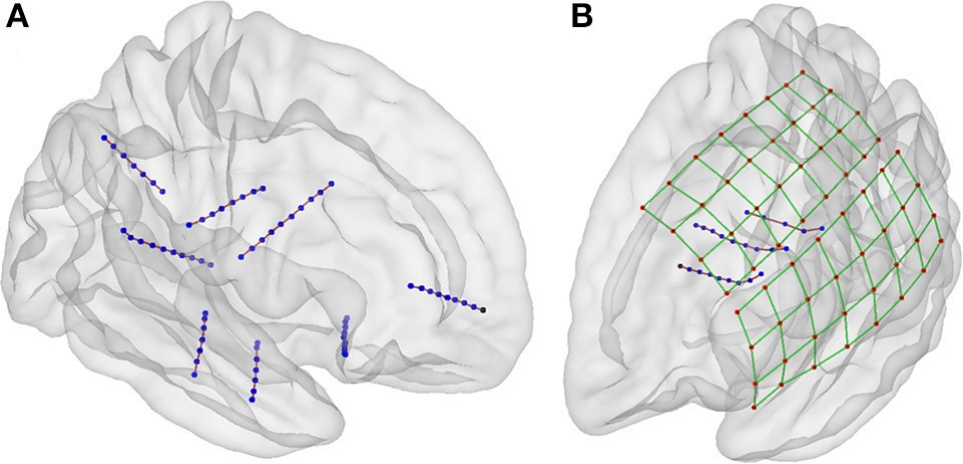
**Localization of electrodes in 3D view. (A)** An example of localized of depth electrodes in a simple scenario, consisting of eight depth electrode arrays (Patient 22). **(B)** Example of localized of depth and grid electrodes in a complex scenario, formed by two grids and three depth electrode arrays entering through the gap between these two (Patient 5). In the last scenario, the selection of voxels in a 3D view is extremely simpler than the selection in 2D views. Additionally, the 3D view of localized coordinates on a rendered semitransparent brain helps to easily understand the relationship between electrodes and brain anatomy.

Some geometrical configurations of electrodes were more complex than others, such as when depth electrodes crossed very closely, when depth electrodes penetrated through grids, or when connection cables lay along electrode grids. In these cases, 3D views were especially useful in determining which CT voxels corresponded to each array. Figure 7 shows the localization results in 3D views for two scenarios, a simple case with only depth electrodes, and a more complex case, with two lateral grids and three depth electrodes entering through the gap between these two grids.

The time required for pre-processing was ~7 h per participant, of which about 95 per cent was used to obtain the brain mask in FreeSurfer. The other processes took on average 15 min: MRI and CT registration ~5 min, MRI and CT normalization ~7 min, MRI brain extraction ~3 min (in a Dell Precision, 8 cores 2.3GHz, 16GB RAM, Dell Inc. USA). Then, electrode localization and electrode labeling in iElectrodes toolbox required 2–3 min per electrode array.

Electrode coordinates were saved in MATLAB and text format files for future use with common EEG analyses toolboxes such as EEGLAB (Delorme and Makeig, 2004), FieldTrip (Oostenveld et al., 2011), and SPM (Litvak et al., 2011).

### Visual validation

During visual inspection, all 1,242 electrodes were located correctly over the corresponding CT artifact in the 2D orthogonal views of the blended MRI-CT images (see Figures 5A–F as an example). Additionally, we verified in the 3D rendered view the numbering of the electrodes.

### Quantitative validation

**Figure 8:**
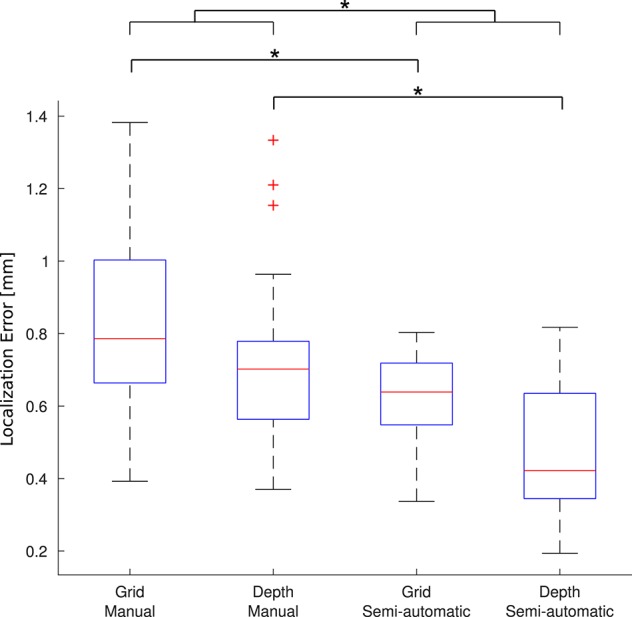
**Localization error**. Each boxplot shows the error (distance to the gold standard) for manual localization and for using iElectrodes to semiautomatically localize grids and depth electrodes. The center lines of each boxplot represents the median and the edges are the 25th (Q1) and 75th (Q3) percentiles. Whiskers are located at Q1 −1.5(Q3 −Q1) and Q3 +1.5(Q3 −Q1), and outliers are plotted outside this interval. ^*^Denotes *p* < 1 × 10^−5^.

A group of 5 experts manually localized a subset of 91 electrodes in 5 patients (number 2, 7, 13, 17, and 21). The experts localized the electrodes both manually and using the toolbox. For each electrode, the mean coordinate from the expert's manual localization was taken as the “gold standard” location to assess the accuracy of the new semi-automatic method as well as the manual method. Krippendorff's alpha, a measure of the inter-rater agreement, was 0.995 for the manual localization coordinates indicating that the experts coordinates were reliable to build a “gold standard.” Overall, the mean localization distance per electrode between the gold standard and the new semi-automatic method was $\bar{e^{s-G}}$ = 0.56 mm ± 0.28 mm, significantly smaller than the manual method $\bar{e^{m-G}}$ = 0.79 mm ± 0.38 mm (*p* < 1 × 10^−5^, permutation paired *t*-test). Also individually, for depth electrodes and grid electrodes, the proposed method achieved smaller localization errors than the manual method: for depth electrodes there was $\bar{e_{depth}^{s-G}}$ = 0.46 mm ± 0.18 mm vs. $\bar{e_{depth}^{m-G}}$ = 0.71 ± 0.21 mm (*p* < 1 × 10^−5^, permutation paired *t*-test), and for grid electrodes there was $\bar{e_{grid}^{s-G}}$ = 0.63 mm ± 0.11 mm vs. $\bar{e_{grid}^{m-G}}$ = 0.83 mm ± 0.25 mm (*p* < 1 × 10^−5^, permutation paired *t*-test). Therefore, the localization using the semi-automatic method showed to be more accurate than the manual one in all cases. Figure 8 shows a box-whisker plot of the localization errors for each category.

The average time reported by experts for manual localization was 49 min (minimum 33, maximum 70 min), whereas it took 22 min (minimum 20, maximum 25 min) to operate the semi-automatic localization. It is important to notice that the manual localization time did not include numbering and anatomical labeling, which were automatically and almost instantaneously obtained with the proposed method.

We investigated the robustness of the proposed method, measuring the distance between the semi-automatic localized electrodes by the individual experts with its mean semi-automatic localization. The variability in localizations was due to thresholding, masking and voxel selection. The mean error distance was $\bar{e^{s-V}}$ = 0.10 mm ± 0.05 mm. Krippendorff's alpha was used as a measure of the inter-rater agreement of the proposed method. Krippendorff's alpha was 0.999 indicating nearly-perfect reliability (Krippendorff, 2004).

### Normalization performance

**Figure 9:**
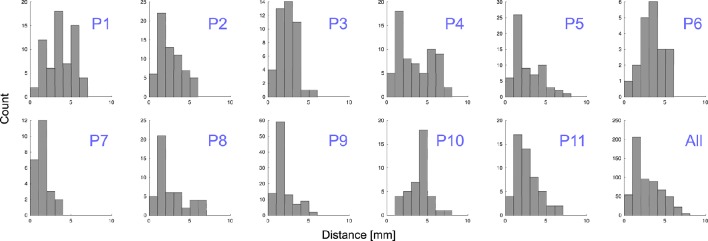
**Distance to MNI cortex**. Histograms showing the estimated distance of subdural grid electrodes to the smoothed cortical envelope (SCE) for each patient, P1–P11, and for all of them together.

Grid electrodes were projected to the SCE while minimizing an energy cost function. The median displacement of grid electrodes (||D_*k*_||) was 2.31 mm ($\bar{D}$ = 2.77 mm ± 1.62 mm) and maximum distance 7.46 mm. When depth and grid electrodes were combined (patients 4–11), a displacement field function was estimated based on the grid electrodes' projection and applied to the depth electrodes. The median displacement of depth electrodes (|| F_*j*_||)) was 0.56 mm ($\bar{\left||F|\right|}$ = 0.81 mm ± 0.81 mm) and a maximum displacement of 3.21 mm. Figure 9 shows the projection distance histograms for each and all patients implanted with grids. No displacement was calculated in patients implanted with deep electrodes only (patients 12–22).

To understand the variability of these distances, we tested and found no correlation between electrodes displacement and either the total number of implanted electrodes or the size of the grid (Spearman's rank correlation test, *p* = 0.97 and *p* = 0.93 respectively). However, we noticed a correlation between the electrodes distance to the SCE and the SCE distance to the pial brain surface (Spearman's rank correlation test, rho = 0.13, *p* = 0.001). In other words, the electrodes located over sulci showed a tendency of longer distances to the SCE (see Supplementary Figure 3).

## Discussion

We have introduced a new method to accurately and semi-automatically localize intracranial electrodes using MRI and CT images. The method returns electrode coordinate locations in standardized MNI space and electrode numbering. The evaluation included 22 patients implanted with a total of 1,242 electrodes, demonstrating that the method is robust with respect to electrode types, diverse implantation configurations, and anatomical regions.

A graphical user interface, iElectrodes, was developed to implement this method as an open-source application. The application allows 2D and 3D views on a rendered semitransparent brain that facilitates a straightforward understanding of electrodes locations in relationship to brain anatomy. The application also provides automatic anatomical labeling by utilizing atlases such as the Harvard-Oxford Structural atlas (Jenkinson et al., 2012). Thus, users are given the neuronal location of the electrodes, which is especially useful for neuroanatomical reference. Since the toolbox is open-source, other atlases can be easily imported. Moreover, parcellation images according to the Desikan-Killiany atlas (Desikan et al., 2006) and obtained from the FreeSurfer processing pipeline are supported by the toolbox. Desikan-Killiany parcellation from pre-implantation images showed to be particular useful for native space analyses in the clinical practice (Princich et al., 2013).

This new method of calculating electrode coordinates from the normalized CT images reduces the time it takes to manually identify electrode locations from MRI and/or CT images. Previous studies show that manual identification at hospitals can take anything from 6 h to 3 days (Princich et al., 2013) and requires an expert in MRI neuroanatomy. In comparison, methods like photograph localization can take 3–5 h (Dalal et al., 2008; Pieters et al., 2013). Automatic localization procedures have been proposed reducing the processing time to 15 min (Sebastiano et al., 2006; Taimouri et al., 2014; Arnulfo et al., 2015). However, these earlier automatic methods are limited. Arnulfo et al. (2015) described a method restricted solely to depth electrodes which requires stereotactic implantation coordinates. Sebastiano et al. (2006) presented an automatic method using spatial filters, but is restricted to detect grid type electrodes only. Taimouri et al. (2014) extended the idea to grid and depth electrodes coordinates, yet the method fails to automatically localize all depth electrodes or distinguish close contacts. Alternatively, Yang et al. (2012) proposed a method based on the selection of only a few electrodes, reducing localization time. However, the technique is limited to grids without curvature. Given the limitations of these alternative methods and even though our proposed method is not fully automatic, it substantially reduces localization time to a few minutes and precisely localizes all implanted depth and subdural grid electrodes. Furthermore, we provided a method to automatically number depth and grid electrode arrays.

Our proposed method relies on the detection of CT signals, which are often used for the localization of electrodes in epilepsy intracranial recordings (Ekstrom et al., 2008; Princich et al., 2013; Arnulfo et al., 2015) and in deep brain stimulation (Hebb and Poliakov, 2009; Lee et al., 2010; Horn and Kühn, 2015). Thus, the manual localization of CT artifacts was considered a gold standard in this study. LaViolette et al. (2011b) validated artifact detection in post-implantation MRI and CT coregistrations and showed a qualitative correspondence with intraoperative images; however they reported differences due to brain compression in the CT and MRI images.

Electrode location inaccuracy may occur using observational methods because the central coordinate of each electrode is subjectively identified from a CT image. Instead, we offer a simple method that identifies the central coordinate by calculating the voxel cluster's center of mass. Krippendorff's alpha and a small distance error ($\bar{e^{s-V}}$) showed that our method is robust to user variations including: a wide range of thresholding values, different numbers of mask erosion or dilation iterations, and electrode voxel selections. Also, it has higher accuracy than the manual localizations done by experts ($\bar{e^{s-G}}$ was significantly smaller than $\bar{e^{m-G}}$).

The accuracy achieved by our method was 0.56 mm in contrast to the gold standard manual localization. Similarly, Arnulfo et al. (2015) showed localization errors of 0.5 mm for depth electrodes using an automated method for detecting CT artifacts. In comparison, for subdural grids, methods coregistering intraoperative photography, MRI and X-ray images showed larger localization errors of ~2 mm (Dalal et al., 2008). Previous methods projecting post-implantation CT coordinates onto pre-implantation MRIs reach mean localization errors in the range of 1.31–3.04 mm (Hermes et al., 2010; Dykstra et al., 2012; Yang et al., 2012; Taimouri et al., 2014). In these cases, the CT is known to be deformed while the MRI is not and localized grid electrodes appear “buried” in the cortex up to 14 mm due to fluid build-up (Dalal et al., 2008; Dykstra et al., 2012). The accumulation of fluid is a dynamic process that occurs while electrodes are implanted in the brain (Studholme et al., 2001; LaViolette et al., 2011a,b). Another factor effecting localization is the brain displacement during surgical procedures, when intraoperative photographs are taken, which can exceed 10 mm with the dominant directional component being associated with gravity, and the mean displacement independent of the size and orientation of cranial opening (Roberts et al., 1998). Moreover, LaViolette et al. (2011a) showed grid electrode mean displacements of 5.4 mm (shear of 4.0 mm) between implantation surgery and cranial reopening. Accordingly, there is no clear evidence that intra-operative photography is more reliable than post-implantation images for localization purposes. Both techniques provide clear localization evidence at the time they are performed, but changes may occur since brain deformation is a dynamic process (Studholme et al., 2001). Additionally, post-implantation images have the advantage of being acquired closer in time to the cognitive task recordings.

When using post-implantation MRI and CT, even though both images account for the same levels of deformation, their coregistration becomes a source of errors (Azarion et al., 2014). Therefore, we applied a maximization of mutual information technique, which has been widely used for cross modality coregistrations (Pluim et al., 2003; LaViolette et al., 2011b; Azarion et al., 2014) and showed sub-voxel accuracy results (Maes et al., 1997).

We have emphasized the potential applications of iEEG to cognitive neuroscience, for which iElectrodes provides a tool to compare recording sites with non-invasive studies and population level analysis. We consider the normalization performance achieved by our method as sufficient for identifying the cortical activations in most cognitive neuroscience research studies, but it could, nevertheless, be subject to improvements (median projection displacement ||D_*k*_|| was 2.31 mm for grids electrodes, and median ||F_*j*_|| was 0.56 mm for depths electrodes). Previously, we showed the relevance of using standardized brain spaces such as MNI to allow comparisons of invasive event related potentials with EEG group analysis (Chennu et al., 2013), or to show convergent evidence of a hierarchical prediction network arising from intracranial and MEG recordings (Phillips et al., 2016). Recently, Kadipasaoglu et al. (2014) demonstrated a new method for group analysis, using a surface-based analysis for iEEG grid recordings but it was not suitable for depth or combined depth and grid recordings. Our localization pipeline, could be combined with this approach to address their limitation. Where peri-operative distortions are large, other methods for normalization may have an advantage (see Klein et al., 2009, for a comparative evaluation of methods) and iElectrodes is compatible with other pre-processing methods such as Advance Normalization Tools (ANTS)—Inside Toolkit (ITK) (Avants et al., 2014) and SPM DARTEL (Ashburner, 2007). We recommend iElectrodes users to utilize the best registration and normalization tools available, and to quantify the pre-processing errors when possible.

Even though several studies showed the safety of post-implantation MRI (Davis et al., 1999; Carmichael et al., 2008) some hospitals do not perform this imaging technique with implanted electrodes. To overcome this limitation we provide the users of our toolbox with the possibility of projecting grids to the SCE surface using the method developed by Dykstra et al. (2012) and then translating the depth electrodes accordingly (Taimouri et al., 2014). It is also possible to use the proposed localization pipeline in the native patient space without normalization. However, we did not test native space localization and cannot comment on whether errors and reliability of the method are different without normalization.

In summary, we provide a new toolbox for the research community that meets the criteria of accurate electrode localization of depth and grid electrodes, offers simplicity and speed of application, reliability, and accessible open-source software.

## Availability and compatibility with other toolboxes

The software project is made available to the scientific community in the form of an open-source MATLAB® toolbox that can be freely downloaded from https://sourceforge.net/projects/ielectrodes/ for research porpoises only under GNU General Public License. We recommend iElectrodes users to run on MATLAB version 2016a or latter. Due to technical limitations in MATLAB's graphical engine, some minor visual failures were observed in older versions. However, we will provide compatibility up to version 2013a for a limited period of time. Additional code is provided in order to facilitate pre-processing and interaction with electro-physiology analysis toolboxes as EEGLAB (Delorme and Makeig, 2004) and FieldTrip (Oostenveld et al., 2011).

## Author contributions

AB wrote the article. AB designed and developed the software. AB and HP contributed to the code. JP acquired the images. AB and HP analyzed the data. JR, TB, CM, and SK supervised this work and wrote the article.

## Funding

This work was supported by Consejo Nacional de Investigaciones Científicas y Técnicas (CONICET) to AB and SK, Agencia Nacional de Promoción Científica y Tecnológica (PIDC 53/2012 and PICT 0775/2012 to AB, JP, SK, and PICT 1232/2014 to CM), Universidad Nacional Arturo Jauretche Investiga 2014 to AB and SK, Comisión de Investigaciones Científicas (CIC) to CHM, Medical Research Council (MC-A060-5PQ30 to JR and a Doctoral Training award to HP), Wellcome Trust (103838 Senior Research Fellowship to JR, Biomedical Research Fellowship; WT093811MA to TB), the James F. McDonnell Foundation 21st Century Science Initiative: Understanding Human Cognition to JR.

### Conflict of interest statement

The authors declare that the research was conducted in the absence of any commercial or financial relationships that could be construed as a potential conflict of interest.
