# Supplementary material for: iElectrodes: A Comprehensive Open-Source Toolbox for Depth and Subdural Grid Electrode Localization
Source: Front Neuroinform. 2017 Mar 2;11:14. doi: 10.3389/fninf.2017.00014 (PMC5333374; doi:10.3389/fninf.2017.00014)
Supplement: Supplementary file 3 [file Presentation1.pdf]

Supplementary Material

iElectrodes: a comprehensive open-source toolbox for SEEG and ECoG electrode localization

Alejandro Blenkmann*, Holly N. Phillips, Juan P. Princich, James B. Rowe, Tristan A. Bekinschtein, Carlos H. Muravchik, Silvia Kochen

*** Correspondence:** Alejandro Blenkmann ablenkmann@gmail.com

1 Supplementary Figures

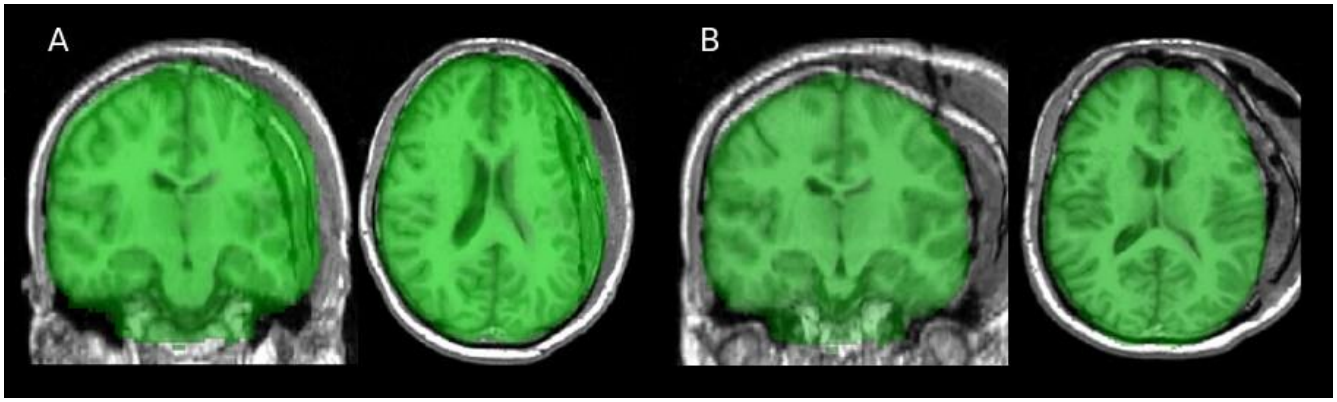

Supplementary Figure 1. Effect of the brain mask in the spatial normalization procedure.

The effect of an individual brain mask for spatial normalization can be observed in the post-normalization images. The MNI average brain mask (green) is overlaid on the MRI images (gray scale). **(A)** Spatial normalization without using brain segmentation mask. Observe that the presence of the grid artifacts and edema in brain regions according to MNI standard. **(B)** Normalization using brain segmentation mask. Notice the grid artifacts closer to MNI brain cortex and non-linear deformation in non-brain areas**.** Example images correspond to patient 2, implanted with an 8x8 grid over the left frontal, temporal, and parietal lobes.

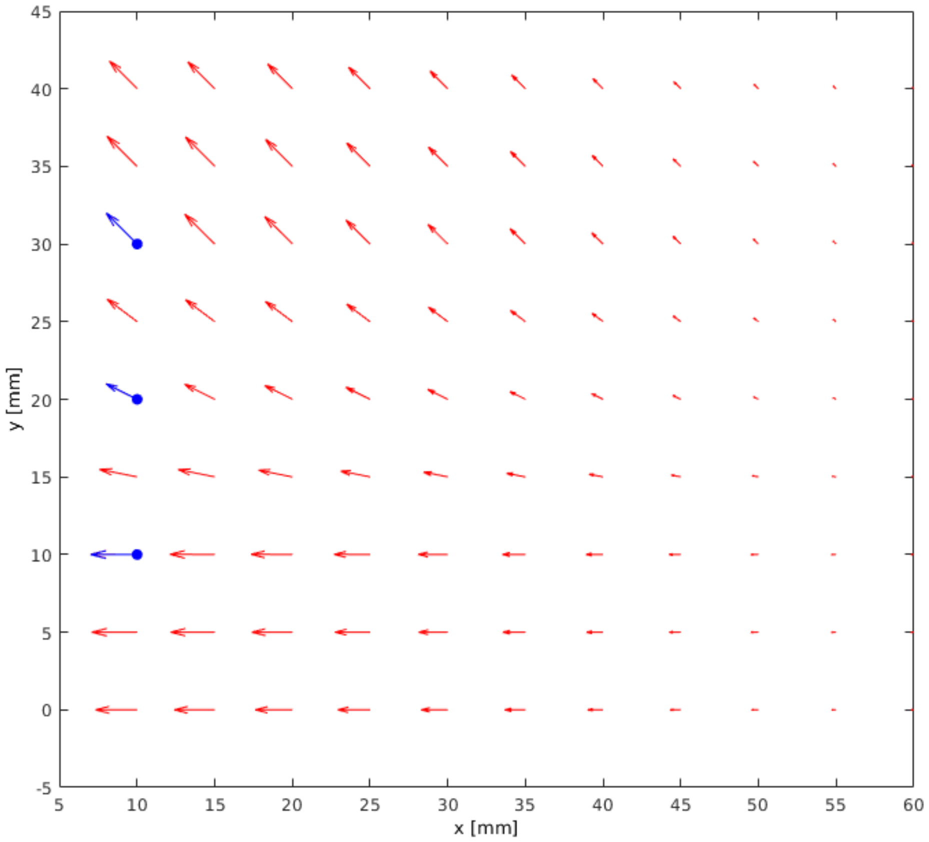

Supplementary Figure 2. Simulated example of the displacement field function.

Three grid electrodes (blue dots) are projected in different directions. The blue arrows represent such projections. The red arrows represent the displacement field function that is applied to correct depth electrode locations. Notice that the displacement strength is attenuated with distance. The displacement field vectors at the grid electrode coordinates are overlapped by the grid displacement. For simplicity, the simulation was done in a 2D space, but the same principle applies for the 3D case. In this example σ_D=30 [mm] and σ_R = 5 [mm].

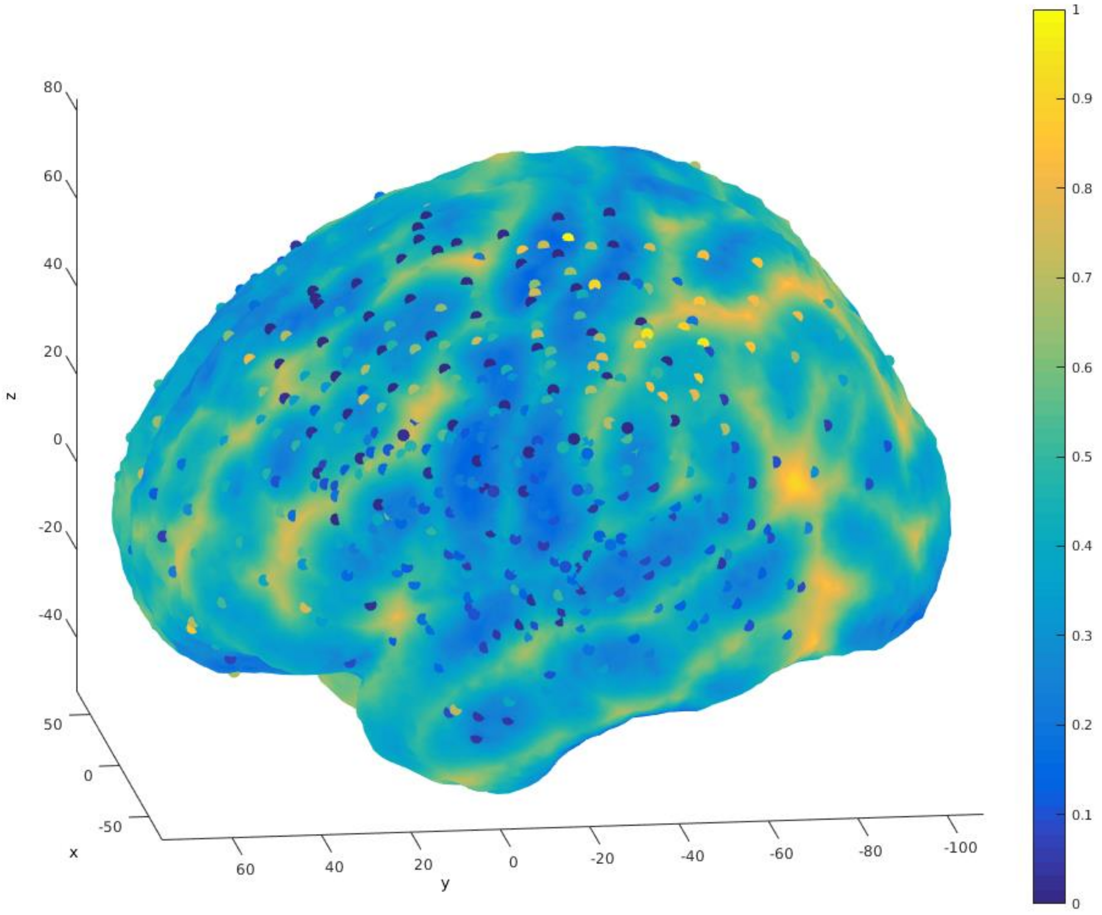

Supplementary Figure 3. Electrode distance to the smoothed cortical envelope (SCE) and the SCE distance to the pial surface. SCE surface is plotted and the color code indicates its distance to the pial surface. Grid electrodes are projected to the SCE surface and their displacement is color coded. Notice the correlation of the most displaced electrodes sitting on top of sulci. More details can be found in section 3.3. Distances were normalized to allow easier comparisons.
